# PTEN Activation by DNA Damage Induces Protective Autophagy in Response to Cucurbitacin B in Hepatocellular Carcinoma Cells

**DOI:** 10.1155/2016/4313204

**Published:** 2016-11-30

**Authors:** Yanan Niu, Wen Sun, Jin-Jian Lu, Dik-Lung Ma, Chung-Hang Leung, Lixia Pei, Xiuping Chen

**Affiliations:** ^1^State Key Laboratory of Quality Research in Chinese Medicine, Institute of Chinese Medical Science, University of Macau, Macau; ^2^Department of Chemistry, Hong Kong Baptist University, Kowloon Tong, Hong Kong; ^3^Longhua Hospital, Shanghai University of Traditional Chinese Medicine, Shanghai 200032, China

## Abstract

Cucurbitacin B (Cuc B), a natural product, induced both protective autophagy and DNA damage mediated by ROS while the detailed mechanisms remain unclear. This study explored the mechanism of Cuc B-induced DNA damage and autophagy. Cuc B decreased cell viability in concentration- and time-dependent manners. Cuc B caused long comet tails and increased expression of *γ*-H_2_AX, phosphorylation of ATM/ATR, and Chk1/Chk2. Cuc B induced autophagy as evidenced by monodansylcadaverine (MDC) staining, increased expression of LC3II, phosphorylated ULK1, and decreased expression of phosphorylated AKT, mTOR. Cuc B induced apoptosis mediated by Bcl-2 family proteins and caspase activation. Furthermore, Cuc B induced ROS formation, which was inhibited by N-acetyl-L-cysteine (NAC). NAC pretreatment dramatically reversed Cuc B-induced DNA damage, autophagy, and apoptosis. Cuc B-induced apoptosis was reversed by NAC but enhanced by 3-methyladenine (3-MA), chloroquine (CQ), and silencing phosphatase and tensin homolog (PTEN). 3-MA and CQ showed no effect on Cuc B-induced DNA damage. In addition, Cuc B increased PTEN phosphorylation and silence PTEN restored Cuc B-induced autophagic protein expressions without affecting DNA damage. In summary, Cuc B induced DNA damage, apoptosis, and protective autophagy mediated by ROS. PTEN activation in response to DNA damage bridged DNA damage and prosurvival autophagy.

## 1. Introduction

Programmed cell death (PCD), a process carried out in a regulated manner, ubiquitously occurs throughout most multicellular organisms' lifespan. To date, three major types of PCD, distinct both morphologically and biochemically, have been established: apoptosis (type I cell death), autophagic cell death (type II), and regulated necrosis (type III) [1–3]. The first and widely investigated type of PCD is apoptosis. Apoptosis is triggered by the activation of cell-surface death receptors by their ligands (the extrinsic pathway) or by induction of the permeabilization of the mitochondrial outer membrane through the Bcl-2 family proapoptotic proteins (Bax, Bak, etc.) (the intrinsic pathway) [1, 4]. Autophagy, a stress response to starvation, acts as an important homeostatic cellular recycling mechanism responsible for degrading unnecessary or dysfunctional cellular organelles and proteins in living cells [5]. Autophagy is characterized by the appearance of large intracellular vesicles and finely controlled by the Atg (autophagy-related gene) family of proteins. In general, it represents a failed attempt to overcome lethal stress and serve as a prosurvival process in response to various stresses. Thus, its function as an active cell death mechanism remains controversial [1]. Actually, most reported autophagy induced by natural products was prosurvival [6, 7]. Regulated necrosis is morphologically characterized by cytoplasmic granulation, organelle and/or cellular swelling resulting from cellular leakage [8].

Accumulated evidence showed that though apoptosis and autophagy were executed through distinct signaling pathways, overlapping signals were engaged in response to specific stimuli [1]. This crosstalk could be mediated by the interactions between Beclin-1 and Bcl-2/Bcl-xL and between FADD and Atg5, caspase- and calpain-mediated cleavage of autophagy-related proteins, and autophagic degradation of caspases [9–13]. Reactive oxygen species (ROS) plays important roles in mediating apoptosis and autophagy in response to a panel of natural products such as evodiamine [14], oridonin [15], graveoline [16], total tanshinones [17], and erianin [18].

Cucurbitacin B (Cuc B), a natural tetracyclic triterpenoid, is abundant in many Cucurbitaceae species [19]. Cuc B induced apoptosis in many cancer line cells [20–25]. The underlying mechanisms include inhibition of JAK/STAT3 [20, 24, 25], induction of DNA damage [23], generation of ROS [26], reduction of G-actin, and activation of cofilin [22]. We firstly reported that Cuc B induced DNA damage mediated by ROS in A549, K562, and MCF-7 cells [23, 27, 28]. Cuc B also induced protective autophagy in HeLa [29], Jurkat [22], MCF-7 [28], and B16F10 cells [30]. Furthermore, Cuc E-, Cuc D-, and Cuc I-induced autophagy was also documented in various cancer cell lines and normal cells [31–35]. Similarly, the underlying mechanisms involve ROS generation and STAT3 inhibition [28, 29, 34, 36]. Interestingly, cucurbitacins-induced autophagy acts as a prosurvival effect [32, 34]. In view of the roles of ROS in Cuc B-induced DNA damage, apoptosis, and protective autophagy, here we reported that Cuc B-induced ROS formation mediated DNA damage, apoptosis, and protective autophagy. The DNA damage activated phosphatase and tensin homolog (PTEN) bridged DNA damage and autophagy.

## 2. Materials and Methods 

### 2.1. Materials and Reagents

Cuc B (>98%) purchased from Chengdu Herbpurify Co., Ltd. (Chengdu, China), was dissolved in dimethyl sulfoxide (DMSO) to make a 100 mM stock solution and was freshly diluted to the desired concentration before use. Primary antibodies for GAPDH, ATM, phosphorylated ATM (p-ATM (Ser1981)), ATR, phosphorylated ATR (p-ATR (Ser428)), Chk1, phosphorylated Chk1 (p-Chk1 (Ser345)), Chk2, phosphorylated Chk2 (p-Chk2 (Thr68)), *γ*-H_2_AX, PTEN, phosphorylated PTEN (p-PTEN (Ser380/Thr382/Thr383)), AKT, phosphorylated AKT (p-AKT (Ser473)), ULK1, phosphorylated ULK1 (p-ULK1 (Ser317)), mTOR, phosphorylated mTOR (p-mTOR (Ser2448)), p62, LC3, Bcl-2, Bik, Bak, cleaved-PARP, cleaved-caspase 7, and cleaved-caspase 9 and secondary antibodies were bought from Cell Signal Technology (Danvers, MA, USA). KU55933 were obtained from Selleck (Houston, TX, USA). Caffeine, monodansylcadaverine (MDC), 3-methyladenine (3-MA), and 5-(6)-carboxy-2′,7′-dichlor-odihydrofluorescein diacetate (DCFH_2_-DA) were purchased from Sigma (St. Louis, MO, USA). N-Acetyl-L-cysteine (NAC) and chloroquine (CQ) were purchased from Beyotime (Haimen, China). Protein phosphatase inhibitor cocktail and propidium iodide (PI) were from Gibco/Thermo Fisher Scientific (Waltham, MA, USA).

### 2.2. Cell Culture

Hepatocellular carcinoma BEL-7402 cells gifted by Professor Jian Ding from the Shanghai Institute of Materia Medica (Shanghai, China) were maintained in a monolayer culture in 95% air and 5% CO_2_ at 37°C in RPMI Medium 1640 (Gibco) supplemented with 10% FBS (Gibco), 100 U/mL penicillin, and 100 *μ*g/mL streptomycin (Invitrogen).

### 2.3. MTT Assay and LDH Release Assay

Cells seeded in 96-well plates for overnight and then treated with or without Cuc B (0–100 nM) for 0–48 h and the cell viabilities were determined by MTT assay. To elucidate the role of ROS, autophagy, DNA damage, and PTEN in Cuc B-induced cell death, cells were pretreated with NAC (2.5 mM), 3-MA (2.5 mM), and CQ (10 *μ*M) or transfected with PTEN siRNA and then cotreated with Cuc B.

LDH release assay was determined by LDH-cytotoxicity assay kit (Beyotime, China) according to the manufacturer's instructions.

### 2.4. Colony Formation Assay

Cells seeded in 6-well plates were treated with or without Cuc B (5–20 nM) and the colony formation assay was performed as in our previous report [27].

### 2.5. Comet Assay

Comet assay was performed as in previous report with minor revisions [37]. Briefly, microscopic slides were coated with 1% normal agarose (GE Healthcare) followed by adding 1% low melting point (LMP) agarose onto each slide and then covering slides with coverslips. The cell suspensions mixed with 1% LMP agarose (1 : 1) were pipetted onto agarose-coated slips. After cooling down, the coverslips were removed and the slides were lowered into freshly made prechilled lysis buffer (2.5 M NaCl, 100 mM EDTA, 10 mM Tris, 1% Triton X-100, and pH 10) for 1 h. Then set the power voltage to 25 V and adjust the current to 300 mA for 20 min to perform the electrophoresis procedure. Cells were stained with PI. Individual cells were viewed using Olympus IX73 fluorescence microscope.

### 2.6. Western Blotting

Treated cells were washed with PBS twice and then harvested using ice-cold RIPA lysis buffer containing protease inhibitor PMSF (Gibco) and protein phosphatase inhibitor cocktail (Gibco). The lysates were centrifuged at 12,500 g for 20 min at 4°C and the supernatant fractions were collected. Protein concentrations were measured with BCA Protein Assay Kit (Gibco). After denaturation at 95°C for 10 min, equivalent aliquots of protein samples (30 *μ*g) were loaded and electrophoresed on SDS-PAGE gels and then transferred to PVDF membrane (Thermo Scientific). The membranes were firstly blocked with 5% nonfat dry milk for 2 h at room temperature and then incubated with primary antibodies (1 : 3000) overnight at 4°C. Then HRP-linked secondary antibodies (1 : 5000) were incubated for 4 h at room temperature. The bands were visualized with the ChemiDoc™ MP Imaging System (Bio-Rad).

### 2.7. MDC Staining

MDC staining used to detect the formation of acidic vesicular organelles in Cuc B-treated cells was performed as in our previous reports [28, 34].

### 2.8. Measurement of Intracellular ROS

The effect of Cuc B on ROS formation was determined as in our previous reports [27, 38].

### 2.9. siRNA Transfection

The siRNA transfection was performed as in our previous report [27]. The sequences of siRNAs were as follows: siRNA sequences for ATM: 5′-GGGCAAUAUUUCAAA UUAATT-3′, 5′-UUAAUUUGAAAUAUUGCC CTT-3′; siRNA sequences for Chk1: 5′-GCGUGCCGUAGACUGUCCATT-3′, 5′-UGGACAGUCUACGGCACGCTT-3′; siRNA sequences for PTEN: 5′-CAGCCGUUCGGAGGAUUAUUCGUCUTT-3′, 5′-AGACGAAUAAUCCUCCGAACGGCUGTT-3′; negative control (NC): 5′-UUCUCCGAACGUGUCACGUTT-3′, 5′-ACGUGACACGUUCGGAGAATT-3′.

### 2.10. Apoptosis Assay

The apoptosis rates after treatment with Cuc B for 6 h were determined by Annexin V/PI double staining by flow cytometry (BD FACSCanto).

### 2.11. Hoechst 33342 Staining

The condensation of DNA was detected by Hoechst 33342 staining as in our previous report [39].

### 2.12. Statistical Analysis

Data were expressed as the means ± SEM from at least three separate experiments performed in triplicate. The differences between groups were analyzed using Prism 5.0 (GraphPad Software Inc., San Diego, CA) and *p* < 0.05 is considered statistically significant.

## 3. Results

### 3.1. Cuc B Inhibited Cells Proliferation

**Figure 1:**
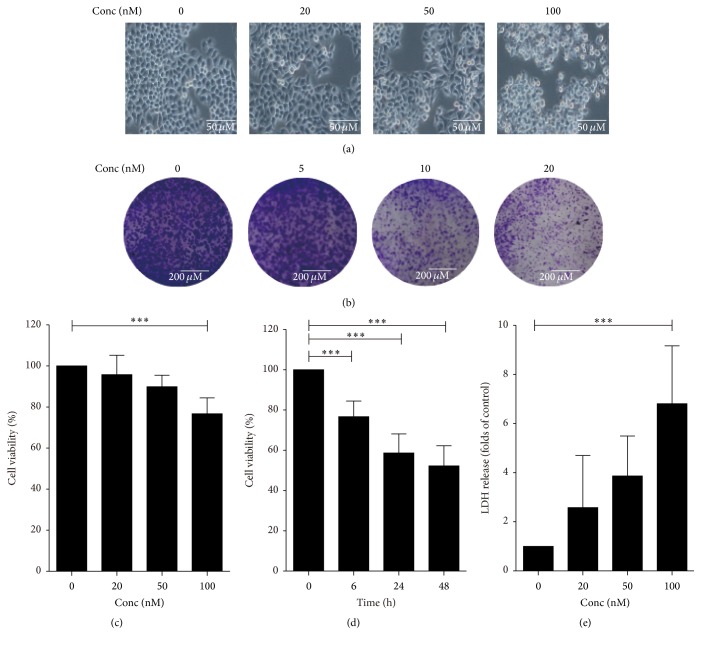
The cytotoxicity of Cuc B to BEL-7402 cells. Cells were treated with Cuc B for 6 h and the cell morphology was observed with microscopy (20x) (a), the cell viability was determined by MTT (c), and the LDH release was determined (e). Cells (6 × 10^2^) were treated with Cuc B for 6 h and then cultured for 2 weeks. The culture medium was replaced every 3 days. The colony was determined by staining with crystal violet and images were captured with a microscope (4x) (b). Cells were treated with Cuc B (100 nM) for 6 h, 24 h, and 48 h and the cell viability was determined by MTT (d). Cuc B, Cucurbitacin B. ^*∗∗∗*^ *p* < 0.001.

Compared with control, morphological alterations were observed in Cuc B-treated cells, especially in 100 nM Cuc B-treated cells (Figure 1(a)). MTT assay showed that Cuc B inhibited BEL-7402 cell proliferation in dose- (Figure 1(c)) and time-dependent manner (Figure 1(d)). Furthermore, Cuc B induced increase of LDH release in the culture medium (Figure 1(e)). In addition, Cuc B dramatically suppressed the colony formation in a dose-dependent manner (Figure 1(b)).

### 3.2. Cuc B-Induced DNA Damage Activated ATM and ATR

**Figure 2:**
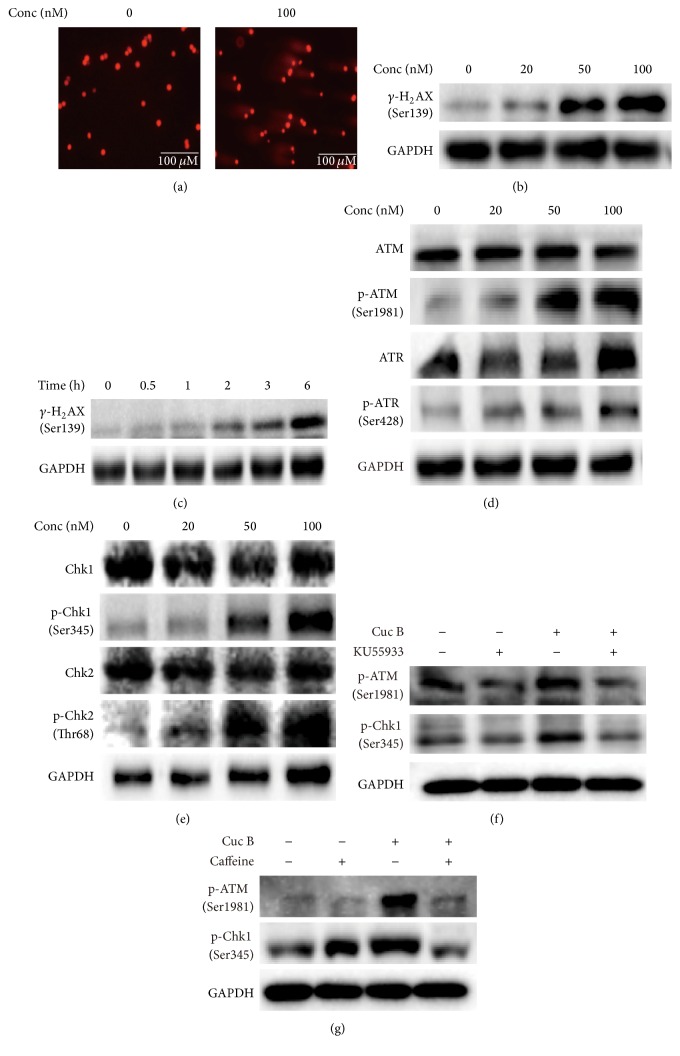
Cuc B induced DNA damage response. Cells were treated with Cuc B for 6 h and the DNA damage was detected by comet assay (a) and the levels of *γ*-H_2_AX (b), ATM, p-ATM, ATR, p-ATR (d), and Chk1, p-Chk1, Chk2, and p-Chk2 (e) were detected by Western blot. Cells were treated with Cuc B (100 nM) and the expression of *γ*-H_2_AX was detected (c). Cells were pretreated with KU55933 (10 *μ*M) (f) or caffeine (10 *μ*M) (g) for 2 h followed by cotreatment with Cuc B (100 nM) for 6 h, and the protein expression was determined by Western blot. Cuc B, Cucurbitacin B.

The comet assay showed that significant long tails were observed in Cuc B-treated cells (Figure 2(a)) indicating the occurrence of DNA damage. Cuc B induced *γ*-H_2_AX expression in dose- (Figure 2(b)) and time-dependent manner (Figure 2(c)). Furthermore, the phosphorylation of both ATM/ATR and Chk1/Chk2 was increased in a dose-dependent manner (Figures 2(d) and 2(e)). In addition, Cuc B-induced p-Chk1 and p-ATM were downregulated by both KU55933 (Figure 2(f)) and caffeine (Figure 2(g)).

### 3.3. Cuc B Induced Protective Autophagy

**Figure 3:**
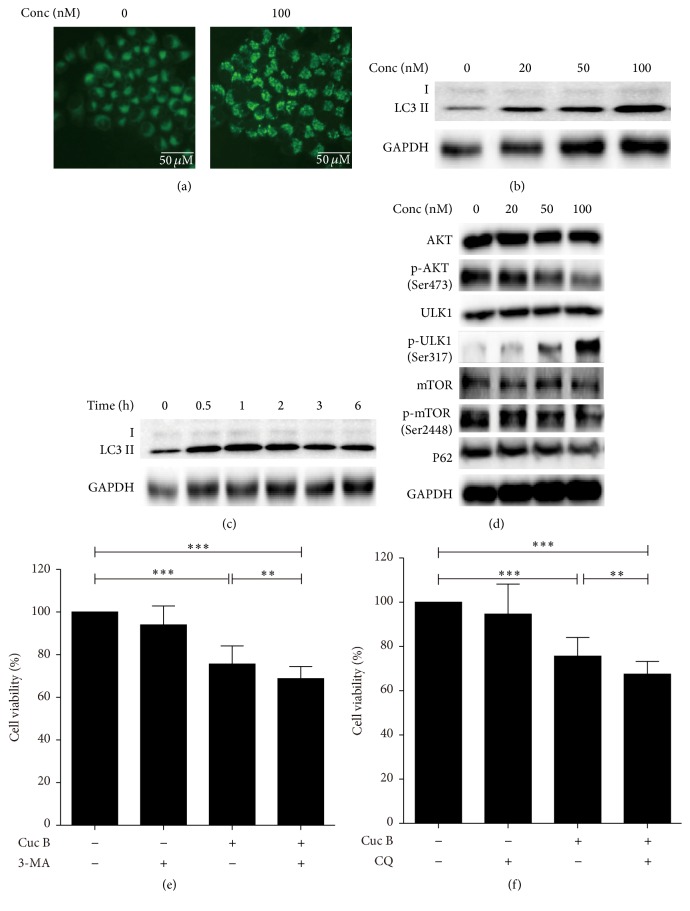
Cuc B induced protective autophagy. Cells were treated with Cuc B for 6 h and stained with MDC (20x) (a) and the protein expression was detected by Western blot (b and d). Cells were treated with Cuc B (100 nM) and the expression of LC3 was detected by Western blot (c). Cells were pretreated with 3-MA (2.5 mM) (e) or CQ (10 *μ*M) (f) for 2 h and then cotreated with Cuc B for 6 h, and the cell viability was determined by MTT. Cuc B, Cucurbitacin B; MDC, monodansylcadaverine. ^*∗∗*^ *p* < 0.01; ^*∗∗∗*^ *p* < 0.001.

Compared with the control group, Cuc B-treated cells showed dramatic increase of intensive green fluorescence in MDC staining suggesting the accumulation of autophagic vacuoles (Figure 3(a)). Cuc B treatment led to upregulation of LC3II/LC3I expression in dose- (Figure 3(b)) and time-dependent manner (Figure 3(c)). Furthermore, Cuc B treatment decreased the expressions of p-mTOR, p-AKT, and p62 and increased expressions of p-ULK1 without affecting total AKT and ULK1 (Figure 3(d)). In addition, 3-MA (Figure 3(e)) and CQ (Figure 3(f)) pretreatment further increased Cuc B-induced cell death.

### 3.4. Cuc B Induced Caspase-Mediated Apoptosis

**Figure 4:**
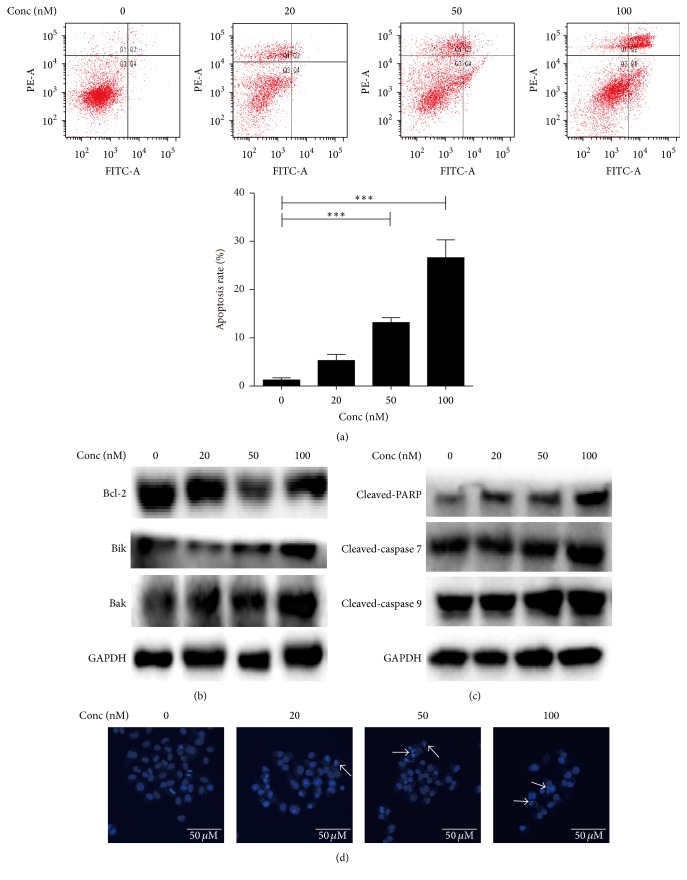
Cuc B induced caspase-mediated apoptosis. Cells were treated with Cuc B for 6 h and apoptosis, protein expressions, and DNA condensation were determined by Annexin V/PI double staining (a), Western blot (b and c), and Hoechst 33342 staining (20x) (d), respectively. Cuc B, Cucurbitacin B. ^*∗∗∗*^ *p* < 0.001.

Cuc B induced apoptosis in a dose-dependent manner (Figure 4(a)). Furthermore, the expression of proapoptotic Bik and Bak was increased while the expression of antiapoptotic protein Bcl-2 was slightly decreased (Figure 4(b)). Increased cleavage of caspase 9, caspase 7, and PARP was also observed (Figure 4(c)). In addition, condensed chromatin was observed in Hoechst 33342 staining after Cuc B treatment (Figure 4(d)).

### 3.5. Cuc B-Induced ROS Formation Resulted in Cell Death

**Figure 5:**
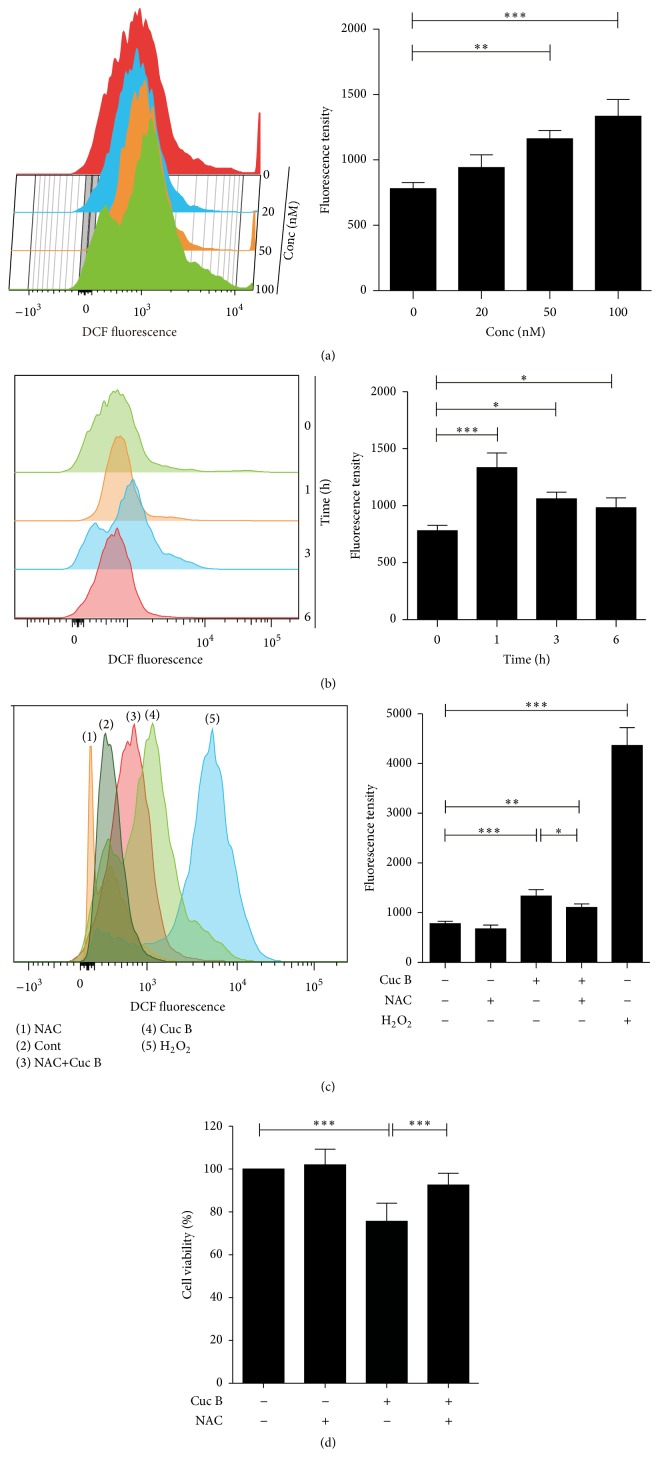
Cuc B induced ROS formation. Cells were treated with Cuc B for 1 h and the ROS generation was detected with DCFH_2_-DA (a). Cells were treated with Cuc B (100 nM) and the ROS formation was detected (b). Cells were pretreated with NAC (2.5 mM) for 1 h followed by cotreatment with Cuc B for 6 h and the ROS (c) and cell viability were determined (d). H_2_O_2_ (1 mM), positive control. Cuc B, Cucurbitacin B; ROS, reactive oxygen species. ^*∗*^ *p* < 0.05; ^*∗∗*^ *p* < 0.01; ^*∗∗∗*^ *p* < 0.001.

Cuc B significantly induced ROS formation in a dose-dependent manner after 1 h treatment (Figure 5(a)), which was dramatically decreased at 6 h (Figure 5(b)). NAC pretreatment decreased Cuc B-induced ROS formation (Figure 5(c)) and reversed Cuc B-induced cell death as well (Figure 5(d)).

### 3.6. ROS Mediated Cuc B-Induced DNA Damage, Autophagy, and Apoptosis

**Figure 6:**
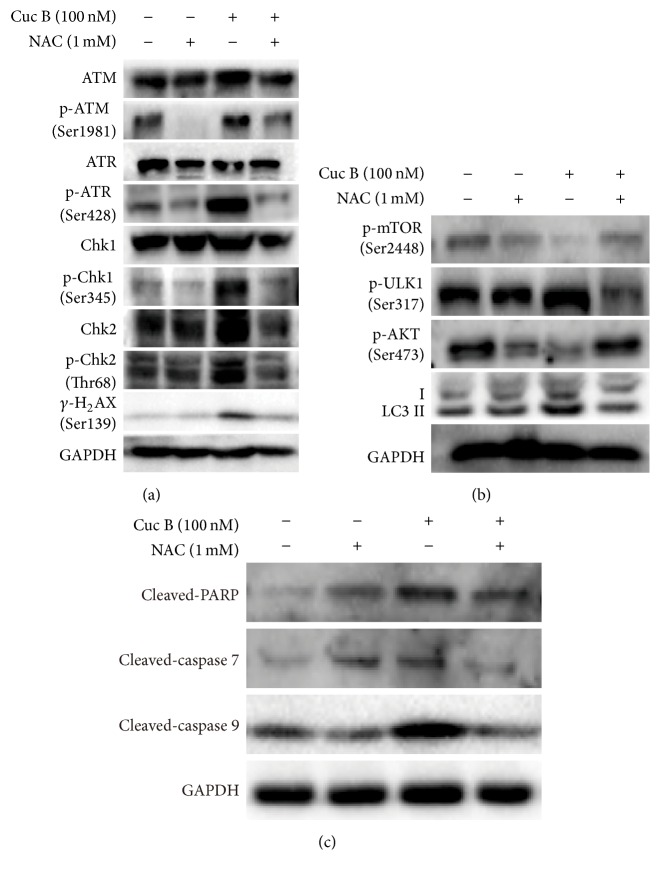
ROS mediated Cuc B-induced DNA damage, autophagy, and apoptosis. Cells were pretreated with NAC (2.5 mM) for 1 h followed by cotreatment with Cuc B for 6 h, and the expressions of DNA damage related proteins (a), autophagy-related proteins (b), and apoptosis related proteins (c) were detected by Western blot. Cuc B, Cucurbitacin B; NAC, N-acetyl-L-cysteine.

The roles of ROS in Cuc B-induced DNA damage, autophagy, and apoptosis were further examined. Cuc B-induced expression of DNA damage response related proteins, *γ*-H_2_AX, and phosphorylation of ATM and ATR and Chk1 and Chk2 were significantly decreased by NAC pretreatment (Figure 6(a)). Furthermore, the deregulated autophagy-related proteins expression such as decreased expression of p-mTOR and p-AKT and increased expression of p-ULK1 and LC3II was also reversed by NAC (Figure 6(b)). NAC alone also decreased expression of p-AKT. In addition, the cleaved caspases and PARP were reversed by NAC pretreatment (Figure 6(c)).

### 3.7. DNA Damage Mediated Autophagy in Response to Cuc B

**Figure 7:**
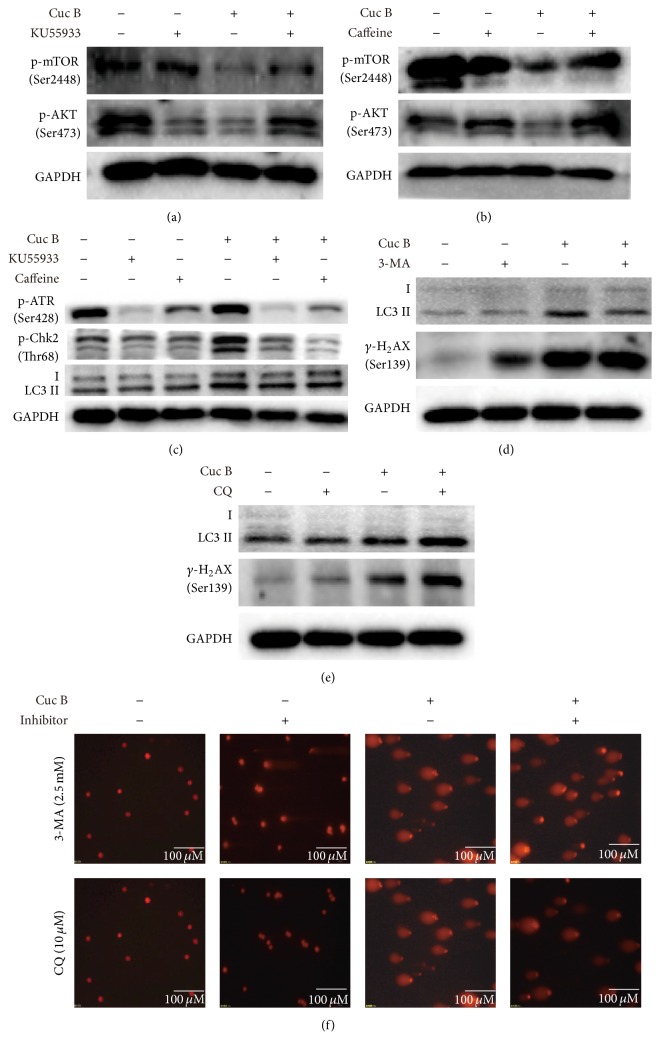
DNA damage mediated autophagy in response to Cuc B. Cells were treated with KU55933 (10 *μ*M) or caffeine (10 *μ*M) for 2 h followed by cotreatment with Cuc B for 6 h and the expression of proteins (a–c) was detected by Western blot. Cells were pretreated with 3-MA (2.5 mM) or CQ (10 *μ*M) for 2 h and then cotreated with Cuc B for 6 h and the protein expression and DNA damage were detected by Western blot (d and e) and comet assay (10x) (f), respectively. Cuc B, Cucurbitacin B; 3-MA, 3-methyladenine; CQ, chloroquine.

Since both DNA damage and autophagy were initiated by Cuc B, their relationship was clarified by applying DNA damage response inhibitors KU55933 and caffeine and autophagy inhibitors 3-MA and CQ. KU55933 and caffeine reversed Cuc B-induced decrease of p-mTOR and p-AKT (Figures 7(a) and 7(b)). They also reversed Cuc B-induced LC3II, p-ATR, and p-Chk2 (Figure 7(c)). However, 3-MA and CQ showed no effect on *γ*-H_2_AX expression (Figures 7(d) and 7(e)). In addition, the long tails caused by Cuc B were not affected by either 3-MA or CQ (Figure 7(f)).

### 3.8. Cuc B Increased PTEN Expression

**Figure 8:**
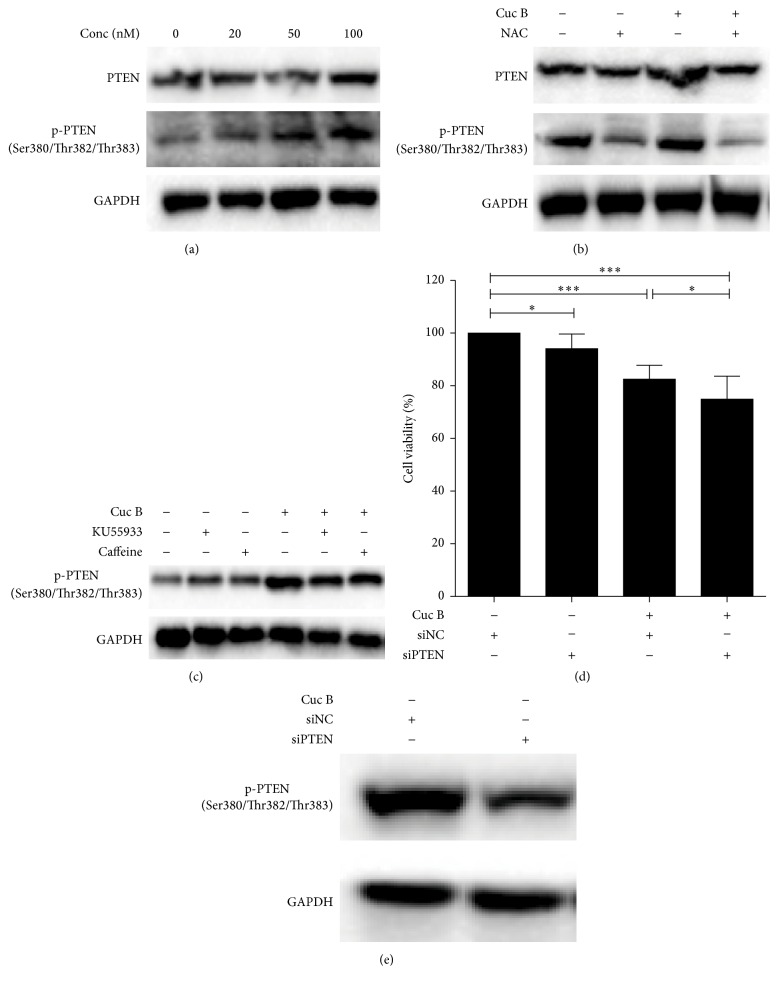
Cuc B activated PTEN expression. Cells were treated with Cuc B for 6 h and expression of PTEN was detected by Western blot (a). Cells were pretreated with NAC (2.5 mM) for 1 h followed by cotreatment with Cuc B for 6 h, and the PTEN expression was detected (b). Cells were pretreated with KU55933 (10 *μ*M) or caffeine (10 *μ*M) for 2 h followed by cotreatment with Cuc B for 6 h and PTEN expression was detected (c). PTEN silenced cells were treated with Cuc B for 6 h and the cell viability was determined by MTT assay (d). siRNA for PTEN was transfected into cells for 48 h and the protein expression of p-PTEN was detected by Western blot (e). Cuc B, Cucurbitacin B. ^*∗*^ *p* < 0.05; ^*∗∗∗*^ *p* < 0.001.

Phosphorylation of PTEN was obviously upregulated by Cuc B in a dose-dependent manner (Figure 8(a)). Increased p-PTEN in response to Cuc B could be inhibited by NAC (Figure 8(b)), KU55933, and caffeine (Figure 8(c)). Interestingly, NAC alone decreased p-PTEN while KU55933 or caffeine alone slightly increased p-PTEN (Figure 8(c)). Furthermore, when PTEN was silenced (Figure 8(e)), Cuc B-induced cell death was further enhanced (Figure 8(d)).

### 3.9. PTEN Bridged DNA Damage and Autophagy in Response to Cuc B

**Figure 9:**
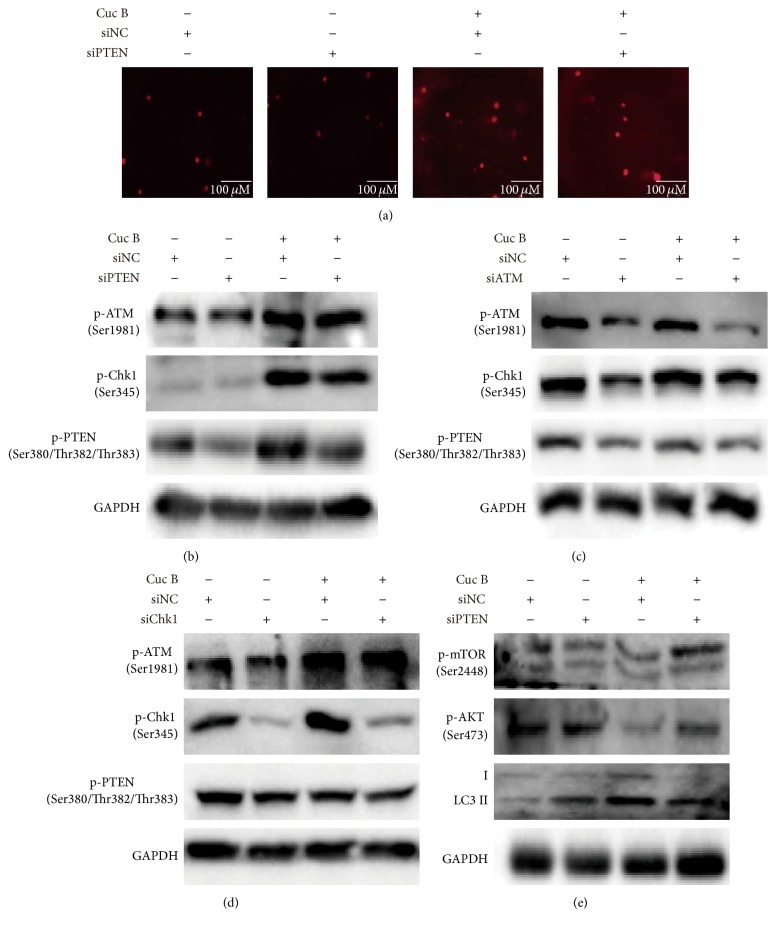
PTEN activation by DNA damage mediated autophagy in response to Cuc B. PTEN was silenced followed by treatment with Cuc B for 6 h and the DNA damage and protein expression were determined by comet assay (a) and Western blot (b and e). ATM or Chk1 was silenced followed by treatment with Cuc B for 6 h and the protein expression was determined by Western blot (c and d).

To dissect the role of PTEN in Cuc B-induced DNA damage, comet assay was performed. PTEN silence showed no effect on the lengths of comet tails (Figure 9(a)) and p-ATM expression but significantly decreased p-Chk1 expression in response to Cuc B (Figure 9(b)), while silence ATM dramatically inhibited Cuc B-induced phosphorylation of both PTEN and Chk1 (Figure 9(c)). Silence Chk1 showed no effect on Cuc B-induced p-PTEN and p-ATM (Figure 9(d)). In addition, silence PTEN reversed Cuc B-induced decrease of p-mTOR and p-AKT and increase of LC3II (Figure 9(e)).

## 4. Discussion

We previously reported that Cuc B induced ROS-mediated DNA damage in A549 cells [27]. The main findings of this study include the following: (1) Cuc B induced DNA damage, apoptosis, and protective autophagy in BEL-7402 cells. (2) ROS was the upstream signals initiating these effects. (3) PTEN activated by DNA damage bridged DNA damage and autophagy in response to Cuc B.

Cuc B-induced cell death was well established in many cell lines. The MTT and colony formation results showed that Cuc B decreased cell viability and inhibited proliferation at nano-mol levels. Increased LDH release suggested that Cuc B might affect cell membrane. We previously reported that Cuc B induced DNA damage in A549 [27], K562 [23], and MCF-7 cells [28]. Cuc B induced long tails in comet assay and increased *γ*-H_2_AX expression suggesting that it induced DNA damage in BEL-7402 cells. The key regulators in response to DNA damage are ATM and ATR kinases, which activated Chk1 and Chk2 [40]. The phosphorylation of ATM/ATR and Chk1/Chk2 was increased by Cuc B, which were dramatically inhibited by ATM inhibitor, KU55933 [41], and ATM/ATR inhibitor caffeine [42]. Thus, Cuc B-induced DNA damage response was mediated by ATM/ATR pathways.

Cuc B-induced autophagy was observed in Jurkat [22] and MCF-7 cells [28]. MDC staining for detecting autophagic vacuoles [43] and increased LC3II expression were simple methods for autophagy assay. The AKT/mTOR pathway, especially the mTOR, has been implicated as the central regulator of autophagy in response to natural products [6]. ULK1, a mammalian serine/threonine protein kinase, plays a key role in the initial stages of autophagy by forming a complex with Atg13 and FIP200 to mediate mTOR signaling [44]. Here, Cuc B increased MDC fluorescence, inactivated AKT/mTOR pathway, and upregulated p-ULK1 and LC3II expression, which suggested that Cuc B induced autophagy mediated by AKT/mTOR pathway. Similar results were observed in MCF-7 cells [28]. Autophagy generally acted as a prosurvival role in response to lethal stress. Protective autophagy was reported in Cuc B-treated MCF-7 [28], Cuc E-treated 95D [34], and Cuc I-treated glioblastoma multiforme cells [32]. Cuc B-induced cell death was further enhanced by autophagy inhibitors 3-MA and CQ suggesting that Cuc B induced protective autophagy in BEL-7402 cells.

Induction of apoptosis by Cuc B was documented. Cuc B induced apoptosis in BEL-7402 cells as evidenced by Annexin V/PI double staining and the Hoechst 33342 staining. Furthermore, Cuc B increased the proapoptotic proteins Bak and Bik expression. However, the antiapoptotic protein Bcl-2 was slightly decreased by Cuc B. Thus, Cuc B-induced apoptosis might be mainly through the upregulation of proapoptotic Bcl-2 family proteins. In addition, the increased cleavage of caspase-7, caspase-9, and PARP revealed that apoptosis was caspase-dependent.

Cuc B-induced ROS played important roles in DNA damage, apoptosis, and autophagy [23, 26, 27, 29]. Here, Cuc B-induced ROS formation was also observed in BEL-7402 cells. Furthermore, Cuc B-induced ROS was increased as early as after 1 h treatment suggesting that ROS formation was an early event. NAC dramatically inhibited Cuc B-induced protein expression related to DNA damage, apoptosis, and autophagy. Thus, ROS mediated Cuc B-induced DNA damage, apoptosis, and autophagy in BEL-7402 cells. DNA damage-induced apoptosis has been well recognized while its role in autophagy remains unclear [45]. Here, we found that Cuc B-induced autophagy was inhibited by KU55933 and caffeine while 3-MA and CQ showed no effect on DNA damage. Collectively, the present data suggested that DNA response triggered autophagy in response to Cuc B. It is interesting to note that p-AKT was decreased by NAC treatment. Similar result was reported in oral cancer cells [46]. We considered that Cuc B-induced massive DNA damage stress led to AKT depression while NAC reversed this depression by inhibiting DNA damage through scavenging ROS.

PTEN, a tumor suppressor gene, has been demonstrated to play a critical role in DNA damage repair and DNA damage response [47]. It also opposes PI3K function, negatively regulates PI3K/AKT pathway, and thus leads to inactivation of AKT and mTOR signaling [48]. A recent study showed that Cuc B inhibited SH-SY5Y cells proliferation through upregulation of PTEN [49]. Here, we found that Cuc B increased p-PTEN expression in BEL-7402 cells, which was inhibited by DNA damage inhibitors and NAC suggesting that activation of PTEN was mediated by DNA damage following ROS generation. Silence PTEN showed no effect in comet assay suggesting that PTEN was not involved in Cuc B-induced DNA damage although decreased Chk1 was also observed. Silence ATM decreased Cuc B-induced PTEN expression while silence PTEN did not affect ATM expression, suggesting that ATM activation resulted in PTEN upregulation. Furthermore, silence PTEN reversed Cuc B-induced autophagy-related protein expression suggesting that PTEN was involved in Cuc B-induced protective autophagy. This was further supported by the enhanced cytotoxicity of Cuc B in PTEN silenced cells. These results were consistent with a recent report showing that ATM mediated PTEN phosphorylation and autophagy in response to DNA-damaging agents in A549 cells [36]. Collectively, these results showed that PTEN activation by DNA damage might act as an upstream molecule of autophagy.

**Figure 10:**
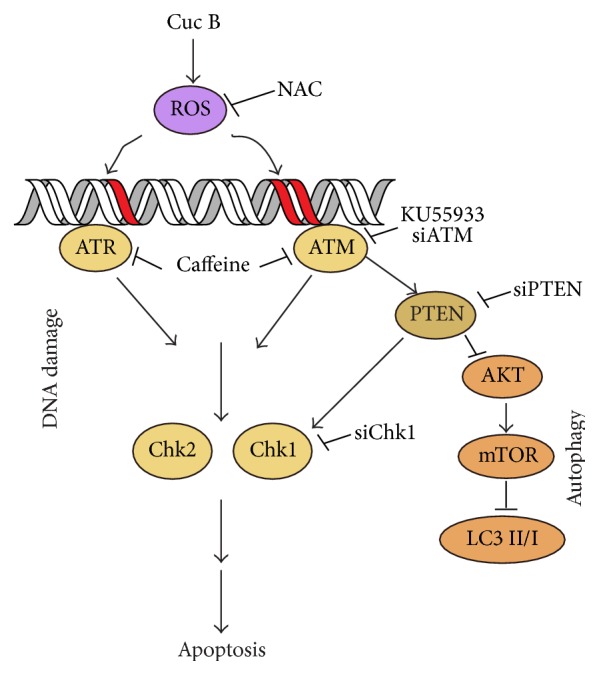
Cuc B induced ROS-mediated DNA damage, apoptosis, and protective autophagy in BEL-7402 cells.

In summary, as depicted in Figure 10, this study showed that a natural product, Cuc B, induced ROS-mediated DNA damage, apoptosis, and protective autophagy. The DNA damage activated PTEN linked the crosstalk between DNA damage and autophagy. This study provides potential roles of PETN in the interplay of prodeath DNA damage and the prosurvival autophagy.

## Abbreviations

ATM: Ataxia-telangiectasia mutated
ATR: ATM and RAD3-related
Chk1: Checkpoint kinase 1
Chk2: Checkpoint kinase 2
Cuc B: Cucurbitacin B
CQ: Chloroquine
DCFH_2_-DA: 5-(6)-Carboxy-2′,7′-dichlor-odihydrofluorescein diacetate
FBS: Fetal bovine serum
LDH: Lactate dehydrogenase
MDC: Monodansylcadaverine
MTT: 3-(4,5-Dimethylthiazol-2-yl)-2,5-diphenyltetrazolium bromide
NAC: N-Acetyl-L-cysteine
3-MA: 3-Methyladenine
NC: Negative control
ROS: Reactive oxygen species
PTEN: Phosphatase and tensin homolog.
